# A bleeding heart: case report and review of pericardial angiosarcoma

**DOI:** 10.4322/acr.2024.488

**Published:** 2024-05-22

**Authors:** Ujjwal Madan, Himil Mahadevia, Parth Sharma, Satya Preetham Gunta, Ossama Tawfik, Karen Fritchie, Julian Magadan

**Affiliations:** 1 University of Missouri Kansas City, Division of Internal Medicine, Kansas City, MO, USA; 2 Medical College of Wisconsin, Division of Cardiology, Milwaukee, WI, USA; 3 MAWD Pathology Group, Cytopathologist, Kansas City, MO, USA; 4 Cleveland Clinic, Department of Pathology, Cleveland, OH, USA; 5 University of Missouri Kansas City, Division of Rheumatology, Kansas City, MO, USA

**Keywords:** Pericardial Effusion, Cardiac Tamponade, Sarcoma, Docetaxel, Gemcitabine

## Abstract

Primary cardiac tumors are rare. The cardiac sarcomas are the most common malignant cardiac tumors. These tumors have a dismal prognosis with an overall median survival of 25 months. Clinical features include dyspnea, arrhythmias, pericardial effusions, heart failure, and sudden cardiac death.

The diagnosis is often challenging. Therefore, the cardiac imaging workup plays a central role in addition to a high clinical suspicion in the setting of atypical presentations that do not respond to standard therapies. The echocardiography, computed tomography, and cardiac MRI are crucial in clinching the diagnosis. Multimodal treatment with surgery, chemotherapy, and radiotherapy has been shown to improve outcomes, as opposed to using either of these modalities alone. We describe the case of a 30-year-old gentleman with COVID-19 infection who developed recurrent hemorrhagic pericardial effusions refractory to standard treatment and was eventually diagnosed as a case of pericardial angiosarcoma after his biopsy revealed the diagnosis and staging was performed using PET–CT–FDG scan. Our case re-emphasizes the importance of considering a malignant etiology early in the course of the disease presentation, especially in recurrent hemorrhagic effusions despite an inflammatory cytologic diagnosis of fluid. It also highlights the place for cardiac CT and MRI to ascertain the location and spread and to plan the further course of treatment. If diagnosed early, the estimated survival time can be prolonged by instituting a multimodal approach.

## INTRODUCTION

Cardiac malignancies are a rare subset of neoplasms, often seen as a feature of metastatic disease rather than a primary neoplasm. The autopsy incidence of primary cardiac tumors ranges between 0.001-.030%, and 75% of these tumors are benign.^1^ Malignant tumors are mostly sarcomas; a small percentage are diagnosed as lymphomas. Histologically, the most common primary cardiac sarcomas are angiosarcomas and undifferentiated sarcomas, and the pericardium is a sporadic source of these angiosarcomas.^1,2^ These tumors are more often encountered in men with a median age of 39-44 years.^3,4^

These tumors can have diverse presentations depending on the tumor histology, anatomic location, and size. These presentations include congestive heart failure, chest pain, pericardial effusions, arrhythmias, and conductional disturbances.^5^ The challenging diagnosis hinges upon cardiac imaging. Echocardiography remains the main tool for the diagnosis of cardiac neoplasms, with increasing utilization of newer techniques such as cardiac computed tomography (CT), cardiac magnetic resonance imaging (MRI), and positron emission tomography/computed tomography with fluorodeoxyglucose (PET–CT–FDG) scan.^6^ Interestingly, echocardiography can detect cardiac masses arising from the atria and ventricles but is unreliable in detecting tumors of pericardial origin. Thus, cardiac CT and MRI are the backbones for the radiological diagnosis of pericardial angiosarcomas, accompanied by pathological and immunohistochemical techniques for confirmation. PET–CT–FDG scan aids in staging the extent of involvement.

These tumors carry a poor prognosis with an overall median survival of 25 months due to barricading factors like diagnostic delay, therapeutic difficulty, local recurrences, and high metastatic potential.^4,5^ Optimal management necessitates a multimodal approach consisting of surgical resection, chemotherapy, and radiotherapy (RT).^5,6^ Patients who undergo complete tumor resection have a better prognosis with increased survival.^7,8^ Herein, we describe an interesting case of a young man with recurrent hemorrhagic pericardial effusions and the diagnostic challenges of finding pericardial angiosarcoma as the lurking culprit.

## CASE REPORT

**Figure 1:**
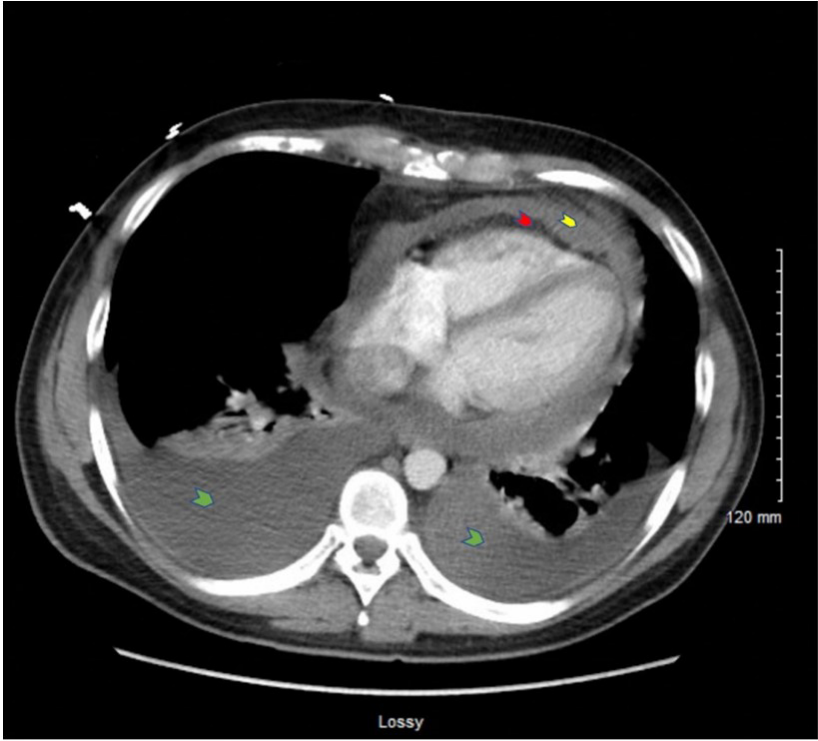
CT chest angiogram demonstrating moderate to large-sized bilateral pleural effusions, greater on the right side (green arrowheads), moderate-sized pericardial effusion (red arrowhead), thickened pericardium (yellow arrowhead), along with mediastinal and hilar adenopathy, and mediastinal fat stranding.

A 30-year-old gentleman with a history of COVID-19 infection one year prior initially presented with 1 week of stabbing chest pain, exertional dyspnea, and abdominal pain. His family and psychosocial history were non-contributory. The physical examination detected distant heart sounds, tachycardia, and jugular venous distension. The transthoracic echocardiogram (TTE) revealed pericardial effusion concerning tamponade physiology. A pericardiocentesis removed 850 cc of bloody effusion, leading to a symptomatic improvement. Workup for autoimmune and infectious causes with cultures, fluid cytology, autoimmune antibody panel (including ANA, anti-smith, anti-DNA, rheumatoid factor, anti-CCP, ANCA serology), HIV, QuantiFERON gold, Bartonella and CMV antibodies were negative. Colchicine and indomethacin were initiated for a presumed viral pericarditis. His symptoms relapsed 20 days later. The thoracic CT was negative for pulmonary embolism but revealed moderate to large pericardial effusion and small bilateral pleural effusions (Figure 1).

The EKG showed sinus tachycardia and electric alternans. The bedside TTE revealed a large circumferential pericardial effusion greater than 2 cm with partial right ventricular diastolic collapse and dilated inferior vena cava. A subxiphoid pericardial window drain placement was performed after the debridement of intrapericardial adhesions. A control TTE revealed an LVEF of 60%, with a moderate circumferential effusion without tamponade. He was continued on indomethacin and colchicine combination.

A few weeks later, the patient experienced dizziness, fatigue, chills, fever, tachycardia, nausea, and hypotension, and the pericardial drain insertion site was tender, with drainage of purulent secretions. He was started on empiric vancomycin and cefepime. Cultures from the pericardial drain grew methicillin-sensitive *Staphylococcus aureus* (MSSA), while the blood cultures were negative. The drain was removed, and the pericardial space MSSA infection was ultimately treated with cefazolin.

**Figure 2:**
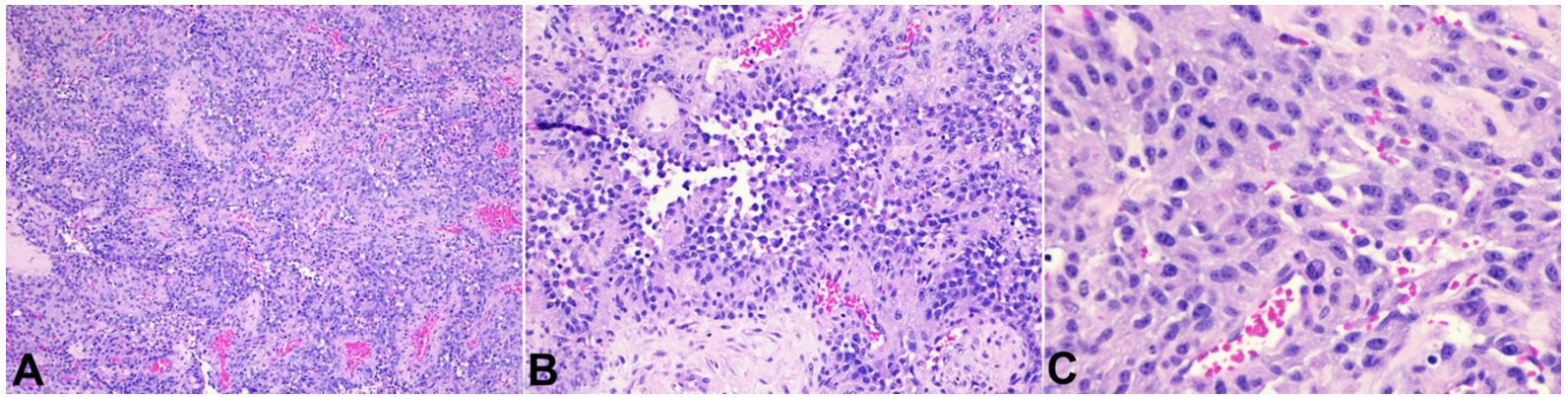
Photomicrograph of the pericardial biopsy. **A –** a low-power view of the malignant epithelioid neoplasm, highlighting the anastomosing pattern of vascular channels (H&E, 10X); **B –** highlights the neoplastic cells with hob-nailing, nuclear enlargement, pleomorphism, and hyperchromasia (H&E, 20X); **C –** highlights nuclear atypia and increase in mitotic activity of the tumor (H&E, 40X).

Shortly thereafter, he started experiencing dyspnea and orthopnea, leg swelling, and non-positional chest discomfort again. Bilateral thoracentesis drained the blood-tinged pleural fluid, and a video-assisted thoracoscopic surgery (VATS) with pleural biopsy was pursued, accompanied by chest tube placement. The pleural specimen pathology was mainly unrevealing. Over time, a sternotomy with pericardiectomy for constrictive pericarditis was indicated, and a pericardial biopsy was performed. The biopsy revealed a malignant epithelioid neoplasm with a formation of anastomosing and dissecting vascular spaces lined by malignant neoplastic cells showing hob-nailing, nuclear enlargement, pleomorphism, and hyperchromasia. Abundant mitotic figures were noted throughout the neoplasm, averaging 25/10 high power fields (Figure 2).

**Figure 3:**
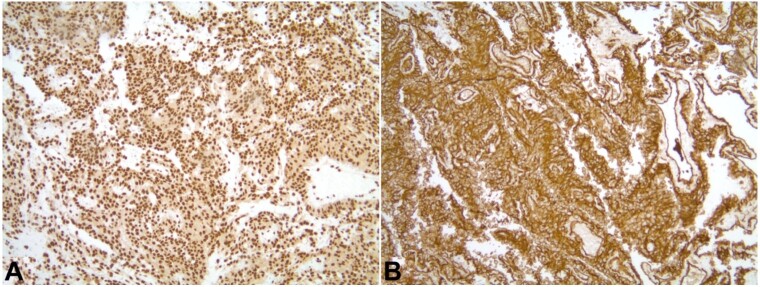
Photomicrograph of immunohistochemistry in low-power view (magnification 10X) with tumor cells showing strong and diffuse staining for ERG (A) and CD 31 (B).

Notably, the neoplastic cells showed diffusely strong positivity for endothelial immunohistochemical stains CD31, ERG, and D2-40 (Figure 3).

The tumor cells were negative for epithelial markers, including pan-cytokeratin, cytokeratin 5/6, CAM 5.2, cytokeratin 7, cytokeratin 20, p40, BER-EP4 and CEA; negative for melanoma markers, including S100, and SOX10; negative for germ cell tumor markers including PLAP, SALL4, CD30, OCT3/4; negative for mesothelial markers including calretinin, and WT-2; negative for neuroendocrine markers including synaptophysin, INSM1. Additional negative stains included CD34, factor XI, Smooth Muscle Actin, and Desmin. These findings were suggestive of a high-grade angiosarcoma involving adjacent lymph nodes. The PET–CT–FDG scan showed hypermetabolic pericardial spaces around the heart and great vessels, consistent with involvement by angiosarcoma, along with pulmonary nodules and intrathoracic lymph nodes consistent with intrathoracic metastases, bilateral nodular pleural thickening, suspicious omental carcinomatosis, and bilateral pleural effusions with loculation and partially collapsed lungs bilaterally. After confirmation of the final diagnosis, the patient was started on chemotherapy with docetaxel and gemcitabine. The CT scan after one month revealed a reduction in pericardial thickening, a significant decrease in multiloculated pleural effusions bilaterally, and an improvement in reticulonodular thickening of mediastinal fat.

## DISCUSSION

Cardiac angiosarcomas are the most common primary malignant cardiac tumors.^9^ They are rare and occur more often in men in the 20-50 age group.^9^ In most cases, the tumor is located in the right atrium.^9^ Pericardial angiosarcoma is occasional.^10^ Symptoms may include fever, weakness, pericardial effusion, heart failure, arrhythmias, and symptoms of metastases.^10^

Our patient initially presented with pericardial bloody effusion with tamponade physiology. Pericardial effusions can have various etiologies, including infectious, autoimmune, inflammatory, drug-induced, and myocardial ischemia. Among these causes, infectious, autoimmune, and malignant etiologies may lead to recurrent pericardial effusions. Interestingly, since the emergence of the COVID–19 pandemic, fatal conditions may initially be misdiagnosed as COVID–19-related complications, resulting in missed opportunities for an early diagnosis of these fatal conditions.^11^ Although our patient had a bloody pericardial effusion, the initial treatment focused on viral etiology, with his history of COVID-19 infection. Multiple TTEs were unremarkable except for pericardial effusion. TTE has 75% sensitivity in detecting cardiac angiosarcomas.^12^ However, TTE often fails to detect tumors located in the pericardium, as in our patient.^12^ Advanced imaging exams like MRI and PET–CT–FDG are usually required.

Furthermore, Li et al.^13^ also described a case of pericardial angiosarcoma presenting as recurring hemorrhagic pericardial effusions, but TTE and pericardial fluid analysis failed to detect the malignancy. Notably, cytological analysis of pericardial fluid has poor sensitivity in discerning pericardial angiosarcoma.^10^ Elevated inflammatory markers and cells in the pericardial fluid may be seen with pericardial angiosarcoma.

Cardiac CT can demonstrate the location, size, and extent of the tumor; however, the cardiac MRI is more sensitive. PET-CT is highly sensitive in detecting pericardial lesions and can demonstrate metastatic disease, thus, additionally aiding in staging the disease and planning further treatment.^13,14^ In our patient, PET–CT–FDG scan showed hypermetabolic pericardial spaces surrounding the heart and great vessels, pulmonary nodules and intrathoracic lymph nodes, nodular pleural thickening, and omental metastasis. Thus, for patients with recurrent pericardial effusions, the cardiac MRI or PET–CT–FDG scan can diagnose this potentially fatal pericardial tumor early. However, pericardial angiosarcoma may remain undetected even with this diagnostic arsenal. Therefore, pericardial biopsy should be considered in patients with unexplained recurrent pericardial effusions and unremarkable advanced imaging studies.

The definitive diagnosis of pericardial angiosarcoma is established by histology and immunohistochemistry (IHC).^10^ In the H&E slides, angiosarcoma often shows abnormal mitosis with epithelioid-shaped, spindle-shaped, and plasmacytic-shaped cells and multiple prominent bar-shaped nucleoli and chromatin strands. IHC staining is also helpful in establishing the diagnosis. In our patient, a pericardial biopsy revealed a malignant epithelioid neoplasm with the formation of anastomosing and dissecting vascular spaces in which the cells showed hobnailing, nuclear enlargement, pleomorphism, and hyperchromasia. These cells were diffusely and strongly positive for CD31, ERG, and D2-40. This was consistent with angiosarcoma.

The therapeutic approach to cardiac angiosarcoma management involves multidisciplinary treatment, including surgery, radiotherapy (RT), and chemotherapy. The median overall survival (OS) of cardiac angiosarcoma is 12 months.^15^ Pericardial angiosarcoma has an overall survival of only 6 months.^15^ It is often diagnosed when it has already metastasized; therefore, the role of surgery is limited. However, combining surgery with chemotherapy may lead to better outcomes if it is diagnosed at an early stage. Timóteo et al.^15^ described a 50-year-old man with localized pericardial angiosarcoma who survived almost 2 years with a combined surgery and chemotherapy approach.

In patients with metastatic cardiac angiosarcoma, the therapeutic cornerstone remains cytotoxic chemotherapy with anthracyclines, ifosfamide, paclitaxel, and docetaxel. Due to a lack of prospective data on neoadjuvant and adjuvant therapy for cardiac sarcomas, the current use of adjuvant therapy for these sarcomas is based on data in the literature on sarcomas involving the trunk and extremities. Anthracycline-based chemotherapy for angiosarcoma demonstrated a median progression-free survival (mPFS) of 4.8 months and overall survival (OS) of 9.9 months.^16^ The Angiotax study evaluated paclitaxel in angiosarcoma and reported mPFS of 4 months, and OS of 8 months.^17^ The GeDDiS trial did not show any significant difference in the proportion of patients alive and progression-free at 24 weeks between the group treated with doxorubicin versus the group of patients treated with a combination of docetaxel and gemcitabine.^18^ Taxanes are shown to have anti-angiogenic effects, which may contribute to their efficacy in angiosarcoma. Our patient was commenced on dose-dense docetaxel and gemcitabine on a 14-day cycle. The chest CT after 1 month of therapy showed partial response. Propranolol was added later to his regime. Banavali et al.^19^ described a 69-year-old female with recurrent metastatic angiosarcoma treated with cyclophosphamide-etoposide and propranolol with a complete response after 2 cycles and a relapse-free survival of 20 months.

Targeted drug therapies like vascular endothelial growth factor (VEGF) antagonists and tyrosine kinase (TK) inhibitors have shown promise and are under evaluation in clinical trials of patients with angiosarcoma. A phase 2 study evaluated bevacizumab in 23 patients with angiosarcoma, with partial response in 2 patients (9%) and stable disease in 11 patients (48%).^20^ A stratified phase 2 study evaluated sorafenib, a VEGFR2, VEGFR3, PDGFR, KIT, and Raf/Mek/Erk pathway inhibitor in angiosarcoma patients. It was divided into two strata. Stratum A included superficial angiosarcoma and Stratum B included visceral angiosarcoma. Progression-free rate (PFR) at 9 months was modest at 3.8% for stratum A and 0% for stratum B.^21^ Sunitinib, an inhibitor of VEGFR1, VEGFR2, VEGFR3, PDGFR, KIT, FLT3, RET, and CSF-1 has shown positive responses in a few case reports.^22,23^ A retrospective study evaluated pazopanib, a multi-targeted TK inhibitor, in advanced vascular sarcomas and showed a 20% response rate in angiosarcoma.^24^ Thus, targeted therapy may benefit patients with advanced cardiac angiosarcoma.

RT in cardiac sarcoma is difficult due to the risk of radiation-induced cardiotoxicity. Furthermore, the heart and lung motion make it difficult to concentrate the beam and can damage the surrounding tissue. However, the development of advanced RT approaches like intensity-modulated radiotherapy (IMRT), breath-holding techniques, and respiratory gating provide some scope to utilize RT. Fields et al.^25^ described a 42-year-old female with right-sided cardiac angiosarcoma who responded to high-dose single fraction RT and was further treated with conformal RT along with concurrent weekly paclitaxel chemotherapy. A combination of chemotherapy with RT using these new technologies may lead to better outcomes in patients with cardiac angiosarcoma.

Evidence is emerging on the role of immunotherapy in the management of angiosarcoma. A patient with metastatic cardiac angiosarcoma treated with a combination of epirubicin, ifosfamide, and pembrolizumab had significant regression of the primary tumor and pulmonary metastases.^26^ A retrospective study evaluated 25 previously treated angiosarcoma patients treated with Pembrolizumab. Pembrolizumab showed a durable clinical response with mPFS of 6.2 months and OS of 72.6 months.^27^ Future prospective studies evaluating targeted therapies and immunotherapy in cardiac angiosarcoma would be valuable, even though the rarity of the disease limits the scope of conducting large-scale studies.

## CONCLUSION

Pericardial angiosarcomas are a rare subset of primary cardiac malignancies that have remained a diagnostic challenge due to the complexity of various presentations described in the literature. It is imperative to keep this diagnosis on the list of differentials, especially in the setting of recurrent hemorrhagic pericardial effusions that have not resolved with the standard therapy and where an etiology has not been established. TTE has a limited role, and cardiac imaging with CT, MRI, and PET–CT–FDG scan should be utilized early in the course of the disease presentation for an early diagnosis and, thus, better outcomes by early intervention with surgery, radiotherapy, or chemotherapy.
